# Pirfenidone Attenuates the EMT Process and the Secretion of VEGF in TGF-*β*2-Induced ARPE-19 Cells via Inhibiting the Activation of the NF-*κ*B/Snail Signaling Pathway

**DOI:** 10.1155/2023/4798071

**Published:** 2023-01-30

**Authors:** Hongsong Li, Lijun Wang, Meilin Shao, Meimei Ren, Wenyi Zhang, Jian Zhou, Jianming Wang

**Affiliations:** ^1^Department of Ophthalmology, The Second Affiliated Hospital of Xi'an Jiaotong University, Xi'an 710004, China; ^2^Department of Ophthalmology, Xijing Hospital, Eye Insitute of PLA, Fourth Military Medical University, Xi'an 710032, Shaanxi, China

## Abstract

**Aim:**

Pirfenidone (PFD), an antifibrotic drug, has various beneficial functions such as antioxidant, antifibrotic, and anti-inflammatory effects. This study aimed to explore the molecular mechanisms underlying how PFD modulates retinal pigment epithelial (RPE) cells involved in neovascularization and subretinal fibrosis.

**Methods:**

ARPE-19 cell lines were treated with transforming growth factor-beta 2 (TGF-*β*2) alone or in combination with PFD. RPE cell viability, as a consequence of PFD use, was determined by the CCK-8 assay. Cell migration was assessed by the wound closure assay and quantified by the Image J software. Protein expression of the following markers was measured by the western blot analysis: an epithelial cell marker and E-cadherin; mesenchymal cell markers, fibronectin, matrix metalloprotein-9 (MMP-9), and alpha-smooth muscle actin (*α*-SMA); a fibrotic marker and connective tissue growth factor (CTGF); an angiogenesis marker and vascular endothelial growth factor (VEGF); NF-*κ*B/Snail. The mRNA levels of fibronectin and *α*-SMA were determined by quantitative real-time PCR. VEGF was quantitatively measured by the enzyme-linked immunosorbent assay.

**Results:**

The cell viability assay revealed that PFD had no significant cytotoxic effect on RPE cells at concentrations of less than 1 mg/mL. The cell scratch assay showed that TGF-*β*2 stimulation significantly improved the migration of RPE cells and that PFD attenuated this effect. PFD significantly inhibited the TGF-*β*2-induced protein expression of E-cadherin and increased the TGF-*β*2-induced protein expression of fibronectin, MMP-9, *α*-SMA, CTGF, and VEGF in ARPE-19 cells. The mRNA expression of fibronectin and *α*-SMA was inhibited by PFD in TGF-*β*2-inducedARPE-19 cells. Additionally, the increased intracellular and supernatant expression of VEGF protein was suppressed by PFD. Mechanistically, RPE cells treated with PFD + TGF-*β*2 exhibited a decrease in phosphorylation of the NF-*κ*B P65 subunit and activation of Snail, compared with the RPE cells treated with TGF-*β*2 alone.

**Conclusion:**

PFD ameliorated TGF-*β*2-induced neovascularization and fibrosis by suppressing the NF-*κ*B/Snail signaling pathway. Therefore, PFD may be a potential drug in the treatment of age-related macular degeneration.

## 1. Introduction

Age-related macular degeneration (AMD) is the most frequent cause of irreversible vision loss among the elderly in developed countries [1]. According to the World Health Organization, the risk of developing AMD will increase with age [2], with an estimated number of 196 million individuals being diagnosed with AMD in 2020 and a predicted number of 288 million individuals being diagnosed in 2040 [3]. AMD is classified into two types: neovascular AMD (“wet” form; nAMD) and non-nAMD (“dry” form), which always result in the impairment of central vision [4]. While nAMD only affects 10%–15% of patients diagnosed with AMD, nAMD accounts for almost 90% of blindness associated with AMD. nAMD is characterized by the presence of choroidal neovascularization (CNV), an ingrowth of abnormal blood vessels from the choroid into the subretinal space, which breaks Bruch's membrane and results in the disruption of the retinal pigment epithelium (RPE) [5].

Recently, intravitreal injection with antivascular endothelial growth factor (VEGF) compounds has emerged as the most effective treatment for nAMD [6]. Although anti-VEGF therapy can maintain and restore macular function to effectively improve patients' visual function, subretinal fibrosis can account for approximately half of all eyes treated within two years after the anti-VEGF treatment [7]. Subretinal fibrosis is regarded as an end-stage condition, which reveals the presence of fibrous plaque and disciform scaring between the neuroretinal and RPE [8]. Previous research has further highlighted the involvement of the epithelial-mesenchymal transition (EMT) of RPE cells in subretinal fibrosis, resulting in the conversion to myofibroblasts that may further advance the fibrosis [9].

Recently, a small synthetic pyridone compound named pirfenidone (PFD) was used as a therapeutic strategy to treat ophthalmologic diseases [10]. PFD (5-methyl-1-phenyl-2-[1H]-pyridone) was first used as an antifibrotic drug for idiopathic pulmonary fibrosis and was shown to have multiple benefits, including antioxidant, antifibrotic activity, and anti-inflammatory effects [11, 12]. The antifibrotic effect of PFD in human pterygium fibroblasts was demonstrated via reduction in the expression of transforming growth factor-*β* (TGF-*β*) and matrix metalloprotein-1 (MMP-1) [13]. PFD reportedly inhibited the viability, migration, and tube formation of human umbilical vein endothelial cells, reduced corneal opacity, decreased the epithelial defect areas, and inhibited the expression of VEGF and the inflammatory reaction after alkali burns [14].

Therefore, this study aimed to evaluate whether PFD suppresses the TGF-*β*2-induced EMT of RPE cells and to further explore the molecular pathways of PFD in CNV and subretinal scar formation.

## 2. Materials and Methods

### 2.1. Cell Culture and Treatments

ARPE-19 cells, an adult RPE cell line (obtained from ATCC), were cultured in a medium (as instructed by ATCC guidelines, containing Dulbecco's modified Eagle medium/F12 (Gibco, USA), 10% fetal bovine serum (Invitrogen, USA)) and stored at 37°C in a humidified 5% CO_2_ incubator. DNA from ARPE-19 cells was extracted and submitted for cell line authentication using a short tandem repeat analysis by Biowing Applied Biotechnology (SBWAB) Co. Ltd. (Shanghai, China) [15]. ARPE-19 cells were divided into four groups according to different treatment methods as follows: control group, PFD group, TGF-*β*2 group, and PFD + TGF-*β*2 group. PFD was purchased from MedChemExpress (cat no. HY-B0673) and reconstituted in sterile water. TGF-*β*2 was purchased from PeproTech (cat no. 100-35B) and solubilized with a recombinant cytokine dissolving and diluent kit (MultiSciences Biotech Co., Ltd, China).

### 2.2. Cell Proliferation Assay

The viability of ARPE-19 cells was determined using a CCK-8 assay (Beyotime, Shanghai, China). ARPE-19 cells were seeded in a 96-well plate at a density of 5 × 10^3^ cells/mL. Cells were treated with 0 mg/mL, 0.1 mg/mL, 0.3 mg/mL, 0.5 mg/mL, and 1.0 mg/mL PFD after being cultured in a serum-free medium for 24 h. After incubation for 48 h, 10 *μ*L of the CCK-8 reagent was added to each well. Subsequently, the absorbance value (*A*) of each sample was detected at 450 nm using a microplate reader (Multiskan, Thermo Fisher Scientific, USA).

### 2.3. Wound Healing Assay and Quantification

ARPE-19 cells were cultured in 6-well plates at a density of 5 × 10^5^ cells/mL.

When cells reached 90%–100% confluence, scratches were made with the head of a 10-*μ*L pipette tip. Cells were then gently rinsed twice with PBS and cultured in a different medium. Images were taken by an inverted microscope (with 40x magnification) at 0 and 48 h after scratching. Wound areas were measured using the Image J (software 1.53e, USA) [16, 17]. Wound closure (%) was quantified using the following formula:

Wound Closure % = [wound area (0 h) − wound area (48 h)/wound area (0 h)] 100%.

### 2.4. Quantitative Real-Time PCR

Total RNA was isolated from the RPE cells with a cell lysis solution (TRIzol; Invitrogen). RNA was reverse transcribed into complementary DNA using a PrimeScript™ RT Master Mix kit (Takara, Japan). According to the manufacturer's instructions, samples were prepared using the TB Green® Premix Ex Taq™ II kit (Takara, Japan) and run on the StepOne Real-Time PCR System (Thermo Fisher Scientific). The sequences of the primers used are as follows: human fibronectin forward 5′-CGGTGGCTGTCAGTCAAAG-3′ and reverse 5′-AAACCTCGGCTTCCTCCATAA-3′; human alpha-smooth muscle actin (*α*-SMA) forward 5′-CCG ACC GAA TGC AGA AGG A-3′ and reverse 5′-ACA GAG TAT TTG CGC TCC GAA-3′; human GAPDH forward 5′-CTCCTC CACCTTTGACGCTG-3′ and reverse 5′-TCCTCT TGTGCT CTTGCTGG-3′. The relative gene expression level was calculated by a previously reported formula [18]:

Relative Quantification (RQ) = 2^−ΔΔCt^.

### 2.5. Western Blotting

The expression of EMT-associated proteins was evaluated by the western blot analysis. Total protein was isolated from RPE cells using the RIPA lysis buffer (Beyotime, Shanghai, China). A BCA assay kit (Beyotime) was used according to the manufacturer's instructions, and protein concentrations of each sample were measured and then normalized to be equally loaded onto SDS-PAGE gels. Following transfer to polyvinylidene fluoride membranes, cell membranes were blocked with 5% nonfat milk for 2 h and then incubated overnight with primary antibodies at 4°C. The antibodies used were as follows: rabbit monoclonal E-cadherin (1 : 1000, CST, USA); fibronectin (1 : 1000, Abcam, UK); matrix metalloprotein-9 (MMP-9) (1 : 1000, Abcam, UK); *α*-SMA (1 : 1000, Abcam, UK); connective tissue growth factor (CTGF) (1 : 1000, Abcam, UK); VEGF (1 : 1000, Abcam, UK); nuclear factor Kappa B (NF-*κ*B) p65 (1 : 1000, CST, USA); NF-*κ*B p-p65 (1 : 1000, CST, USA); snail (1 : 1000, Abcam, UK); and mouse monoclonal GAPDH (1 : 1000, Elabscience, Wuhan, China). PVDF membranes were then incubated with a horseradish peroxidase (HRP)-conjugated goat anti-rabbit/mouse IgG secondary antibody (1 : 10 000, Elabscience, Wuhan, China) at room temperature for 2 h. PVDF membranes were then incubated with the HRP-conjugated goat anti-rabbit/mouse IgG secondary antibody (1 : 10,000, Elabscience, Wuhan, China) at 25°C for 2 h. ImageJ 1.53e (National Institute of Health, USA) was used to quantify the intensity of each band.

### 2.6. Enzyme-Linked Immunosorbent Assay (ELISA)

After each treatment, the levels of VEGF were measured using ELISA. In brief, ARPE-19 cells were cultured in 96-well plates at a density of 5 × 10^3^ cells/mL for 48 h. The supernatant from cell cultures was assayed using the VEGF ELISA kit (Elabscience, Wuhan, China) according to the manufacturer's instructions.

### 2.7. Statistical Analysis

GraphPad Prism 8.02 (GraphPad Software, USA) was used to analyze the data and illustrate the results. Quantitative data were tested for normal distribution using the Shapiro–Wilk test and are presented as mean ± standard deviation (SD) of three independent experiments. Data compared among multiple groups were analyzed using one-way analysis of variance (ANOVA), and the least significant difference analysis was used to evaluate the difference between two groups. *P* < 0.05 was considered statistically significant.

## 3. Results

### 3.1. Effect of PFD on Cell Viability of TGF-*β*2-Induced ARPE-19 Cells

**Figure 1:**
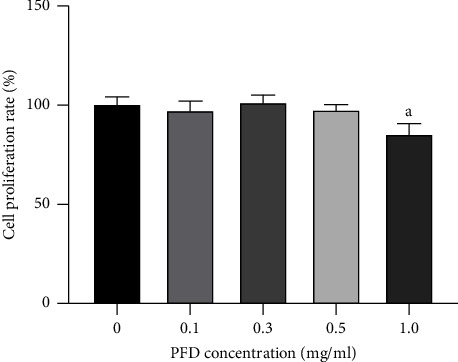
Effect of PFD on cell viability of ARPE-19 cells. ^*a*^*P* < 0.05 vs. 0 mg/mL PFD. PFD: pirfenidone.

The CCK-8 assay was performed in RPE cells treated with different concentrations of PFD. Compared with 0 mg/mL PFD, no toxic effect of PFD was observed at 0.1, 0.3, or 0.5 mg/mL (Figure 1). However, treatment with 1.0 mg/mL PFD had significant effects on cell viability. Based on the results of cell viability, 0.5 mg/mL was used as the concentration of PFD in the subsequent experiments.

### 3.2. PFD Suppressed the Migration of TGF-*β*2-Induced RPE Cells

**Figure 2:**
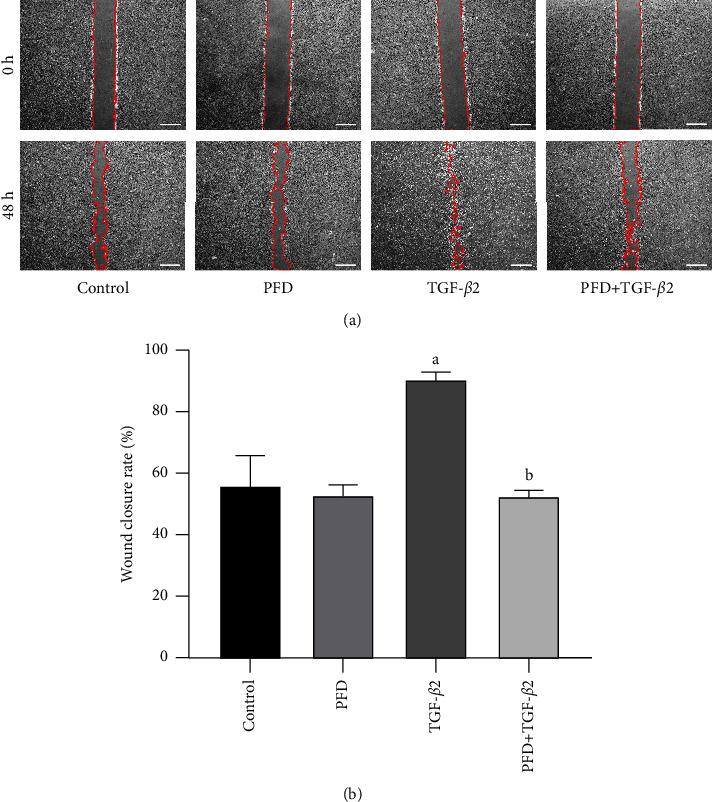
Effect of PFD on the migration of TGF-*β*2-induced RPE cells. (a) Representative images were taken after scratching at 0 h and 48 h. The red line automatically drawn by the ImageJ software indicates the areas without migrating cells. (b) Quantitative analysis of the migration rates in each group is shown in the bar graph. ^*a*^*P* < 0.05 vs. control; ^*b*^*P* < 0.05 vs. TGF-*β*2. PFD: pirfenidone. Scale bar = 500 *μ*m.

Subretinal fibrosis and tissue repair involve a complex process, including RPE proliferation and migration. Therefore, a wound healing assay was performed to explore the effect of PFD on the migration ability of TGF-*β*2-induced RPE cells.  Figure 2(a) reveals that although the cells were cultured in a serum-free medium, cells migrated to varying degrees after 48 h of incubation and when the scratch edges were serrated. Although cells in the PFD + TGF-*β*2 group showed local fusion trends, the wound closure rate was significantly different compared with that in the TGF-*β*2 group (Figure 2(b)). These results confirmed that TGF-*β*2 significantly enhanced RPE migration and that PFD significantly suppressed TGF-*β*2-induced RPE cell migration.

### 3.3. PFD Decreased the Expression of *α*-SMA and Fibronectin in TGF-*β*2-Induced RPE Cells

**Figure 3:**
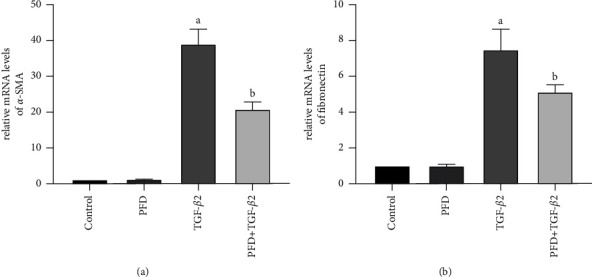
Effect of PFD on the mRNA expression of *α*-SMA (a) and fibronectin (b) in TGF-*β*2-induced RPE cells. ^*a*^*P* < 0.05 vs. control; ^*b*^*P* < 0.05 vs. TGF-*β*2. PFD: pirfenidone.

To further determine the effect of PFD on mRNA levels in EMT, PCR assay was performed. PCR results (Figure 3) revealed that TGF-*β*2 increased the gene expression of *α*-SMA and fibronectin, and this effect was significantly attenuated by PFD. The mRNA levels of *α*-SMA and fibronectin were not significantly different between the control and PFD groups.

### 3.4. Effect of PFD on the Protein Expression of EMT Markers in TGF-*β*2-Induced RPE Cells

**Figure 4:**
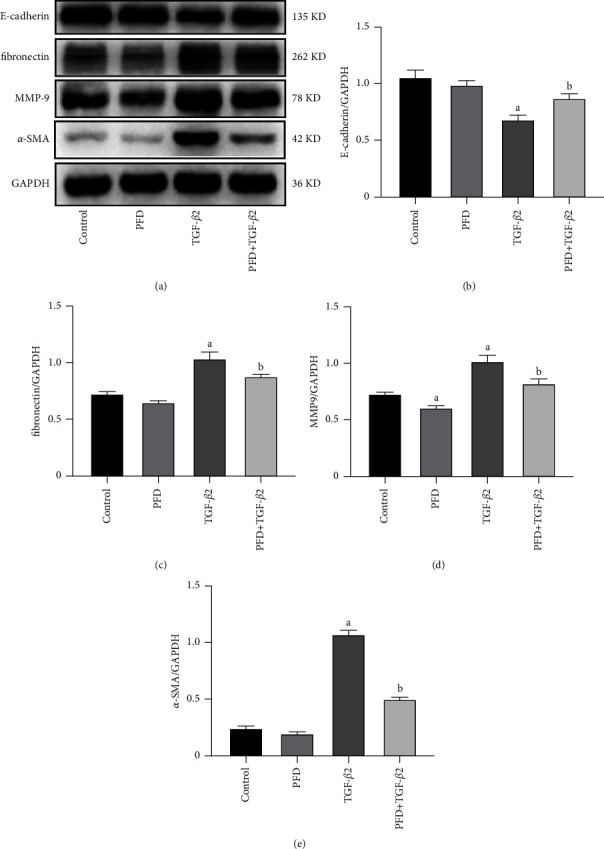
Effect of PFD on the protein expression of EMT markers in TGF-*β*2-induced RPE cells. (a) Protein electrophoretogram; GAPDH was used as a normalization control. Comparison of the relative expression of (b) E-cadherin, (c) fibronectin, (d) MMP-9, and (e) *α*-SMA among different groups. ^*a*^*P* < 0.05 vs. control; ^*b*^*P* < 0.05 vs. TGF-*β*2. PFD: pirfenidone.

The EMT of RPE cells is associated with the development of subretinal fibrosis. Western blot analysis (Figure 4) revealed that the protein expression of the epithelial cell marker E-cadherin was reduced by TGF-*β*2 and that the stimulus was inhibited by PFD. The stimulus of TGF-*β*2 induced an increase in the protein expression of fibronectin, and this change was weakened by PFD. Furthermore, PFD inhibited the elevation of the expression of MMP-9 in TGF-*β*2-induced RPE cells. Stimulation via TGF-*β*2 also increased the expression of *α*-SMA in RPE cells and was attenuated by PFD. Compared with the control group, the PFD group showed no effect on the expression of E-cadherin, fibronectin, and *α*-SMA. However, compared with that noted in the control group, the expression of MMP-9 was inhibited in the PFD group.

### 3.5. PFD Attenuated the Protein Expression of CTGF in TGF-*β*2-Induced RPE Cells

**Figure 5:**
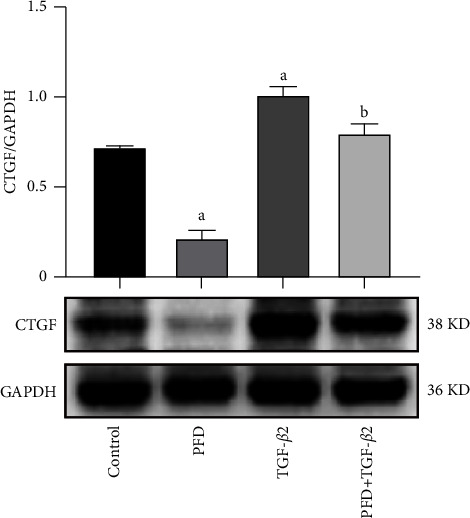
Effect of PFD on the expression of CTGF protein in TGF-*β*2-induced RPE cells. ^*a*^*P* < 0.05 vs. control; ^*b*^*P* < 0.05 vs. TGF-*β*2. PFD: pirfenidone.

Western blot was used to determine the expression of the pro-fibrotic marker CTGF, and the PFD was found to inhibit the stimulation of TGF-*β*2, which markedly attenuated the expression of CTGF in RPE cells (Figure 5). Compared with that noted in the control group, the protein expression of CTGF in the PFD group was also significantly decreased.

### 3.6. PFD Attenuated the Production and Secretion of VEGF in TGF-*β*2-Induced RPE Cells

**Figure 6:**
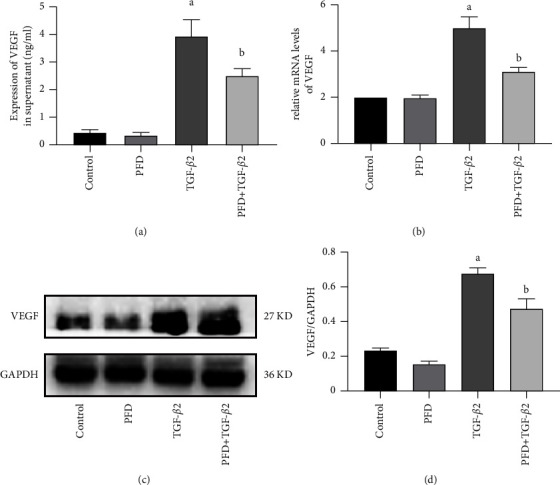
Effect of PFD on the formation and secretion of VEGF in TGF-*β*2-induced RPE cells. (a) Comparison of the relative expression of VEGF in cell culture supernatant among the groups. (b) Comparison of the relative mRNA expression of intracellular VEGF among the groups. (c) Protein electrophoretogram of intracellular VEGF; GAPDH was used as a normalization control. (d) Quantitative comparison of the relative expression of intracellular VEGF protein among the groups. ^*a*^*P* < 0.05 vs. control; ^*b*^*P* < 0.05 vs. TGF-*β*2. PFD: pirfenidone.

To explore the effects of PFD on angiogenesis, the secretion of VEGF into the cell culture supernatant was detected by ELISA, and the protein and mRNA expressions of VEGF were assessed by the western blot analysis and PCR, respectively. ELISA (Figure 6(a)) revealed that TGF-*β*2 increased the secretion levels of VEGF in the cell culture supernatant, and this effect was significantly attenuated by PFD. PCR results (Figure 6(b)) showed that PFD inhibited the increased mRNA expression of intracellular VEGF induced by TGF-*β*2. Similarly, and the western blot analysis (Figures 6(c) and 6(d)) revealed that the protein expression of intracellular VEGF was elevated by TGF-*β*2 induction, and these changes were inhibited by PFD. Overall, PFD inhibited the production and release of intracellular VEGF, which was induced by TGF-*β*2.

### 3.7. PFD Inhibited the Activation of EMT Processes and the Secretion of VEGF via the NF-*κ*B/Snail Signaling Pathway

**Figure 7:**
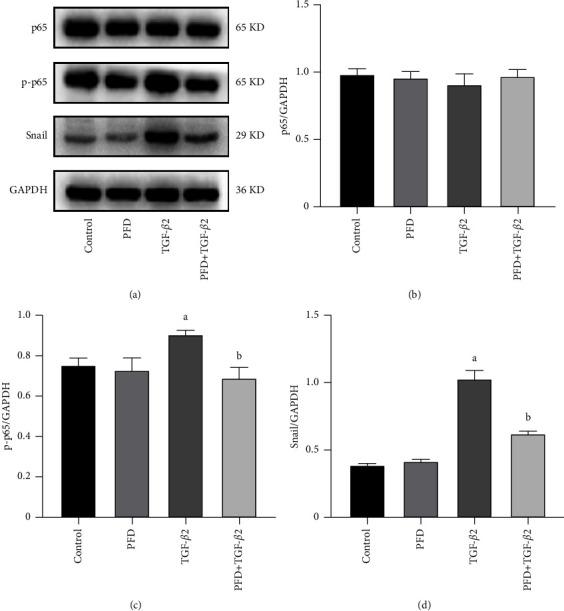
Effect of PFD on TGF-*β*2-induced activation of the NF-*κ*B/snail signaling pathway in RPE cells. (a) Protein electrophoretogram; GAPDH was used as a normalization control. (b) Comparison of the relative expression of p-65 protein among the groups. (c) Comparison of the relative expression of phospho-p65 protein among the groups. (d) Comparison of the relative expression of snail among groups. ^*a*^*P* < 0.05 vs. control; ^*b*^*P* < 0.05 vs. TGF-*β*2. PFD: pirfenidone.

To determine whether the effects of PFD on EMT were mediated by the NF-*κ*B/Snail signaling pathway, the expression of EMT protein upon TGF-*β*2 stimulation was detected. The expression of p-65 was not significantly changed among the groups (Figure 7). Meanwhile, the phosphorylation of p-65, induced by TGF-*β*2 stimulation, was almost completely inhibited by PFD. Similarly, TGF-*β*2 upregulated the expression of Snail, and this trend was reversed by PFD.

## 4. Discussion

This study demonstrated the functional role and underlying molecular mechanisms of PFD in the EMT of RPE cells and its association with angiogenesis and fibrosis. The data obtained herein suggest that PFD suppressed the expression of epithelial markers (E-cadherin) and elevated the expression of mesenchymal markers (fibronectin, MMP-9, and *α*-SMA) in TGF-*β*2-induced RPE cells. Further, the expression of the fibrosis marker CTGF was attenuated by PFD. Interestingly, the secretion of VEGF was partially inhibited by PFD. We further demonstrated that the NF-*κ*B/Snail pathway may play a key role in the EMT process, in the secretion of VEGF and in the formation of CTGF.

Numerous studies have demonstrated that PFD is involved in the treatment of fibrotic diseases, such as idiopathic pulmonary fibrosis and renal fibrosis [19, 20]. PFD is a synthetic pyridine, which has anti-inflammatory, anti-oxidant, and antifibrotic effects, while also being able to inhibit the migration, differentiation, and proliferation of normal RPE cells [21]. Further, PFD has been shown to inhibit the TGF-*β*1-induced expression of ECM via blocking the transduction of phosphorylated Smads [22].

To better model the microenvironment in which RPE cells are located in a diseased state, we chose TGF-*β*2 as an exogenous stimulus to investigate the effect of PFD on RPE cells under pathological conditions. TGF-*β* is a common and evolutionarily conserved secreted protein that induces EMT processes and has three main subtypes: TGF-*β*1 (most common), TGF-*β*2, and TGF-*β*3 [23, 24]. Despite the high degree of homology in their nucleotide sequence, the three proteins exhibit different biological characteristics in different host tissue cells [25]. Co-expression of TGF-*β*1 and TGF-*β*2 isoforms has been reported in the vitreous fluid and pathological tissue sections of human and monkey eyes, with TGF-*β*2 levels being significantly higher than TGF-*β* levels [26, 27]. Therefore, it is necessary to explore the biological changes in RPE cells under TGF-*β*2 stimulation as well as any changes in the EMT process. This study confirmed the facilitating effect of TGF-*β*2 on RPE cell proliferation and established an EMT model induced by TGF-*β*2.

EMT, especially type-2, is a key biological process in wound healing, tissue regeneration, and organ fibrosis that transform the characteristics of epithelial cells into mesenchymal phenotype cells, including the loss of cell-cell and cell-substratum adhesion, and the acquisition of migratory properties [28]. EMT processes are often accompanied by characteristic molecule changes, including the expression of mesenchymal markers (such as *α*-SMA), and a decrease in the expression of epithelial markers (such E-cadherin) and ECM-associated markers (such as MMP-9 and fibronectin) [29]. In this study, the results revealed that the expression of *α*-SMA, MMP-9, and fibronectin was upregulated by TGF-*β*2 in RPE cells in a manner sensitive to inhibition by PFD. Meanwhile, the expression of E-cadherin was downregulated by TGF-*β*2 in RPE cells and was reversed by PFD. These results suggest that EMT alterations, induced by TGF-*β*2, could be effectively attenuated by PFD.

Recently, it was demonstrated that the EMT process and the accumulation of ECM could be mediated by a CTGF-dependent pathway [30]. CTGF, a 38-*k*Da secreted protein, is a prototypic member of the cellular communication network 2 family. CTGF plays an essential role in the regulation of pro-fibrotic and angiogenic factors and is an important marker for fibrosis [31, 32]. Our results demonstrated an increase in the expression of CTGF induced by TGF-*β*2. PFD inhibited the production of CTGF in RPE cells with or without TGF-*β*2 stimulation. The results suggest that PFD could be a potential therapeutic drug for fibrosis diseases. However, further exploration is needed regarding the mechanisms of EMT and the ability of PFD to inhibit fibrosis.

Previous work has demonstrated that the NF-*κ*B subunit, p-65, binds to the Snail promoter and directly upregulates the expression, resulting in the repression of E-cadherin expression [33, 34]. Snail downregulated the expression of other epithelial molecules and induced the expression of genes associated with a mesenchymal and invasive cell phenotype [35]. Furthermore, the fibrotic marker CTGF was identified as a downstream target of Snail. Therefore, the network created between NF-*κ*B and Snail plays a crucial role in the EMT process, the secretion of VEGF, and the fibrosis induced by CTGF. Our study indicated that the NF-*κ*B/Snail signaling pathway was activated in TGF-*β*2-stimulated RPE cells. The activation of the NF-*κ*B/Snail signaling pathway was then attenuated by inhibiting the phosphorylation of p-65. Though the exact relationship between the Snail and VEGF genes remains unclear, our study indicated that NF-*κ*B signaling may act as a mediator of the downstream expression of VEGF.

**Figure 8:**
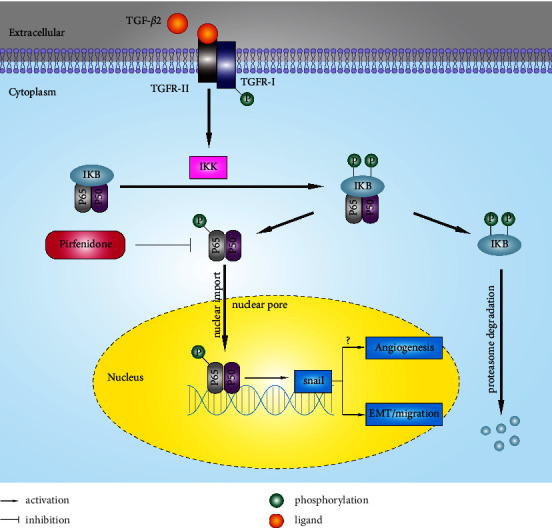
Pirfenidone attenuated the neovascularization and subretinal fibrosis via inhibiting the activation of the NF-*κ*B signaling pathway. TGFR: TGF receptor; IKB: I-kappa B; IKK: IKB kinase.

In conclusion, PFD probably attenuated neovascularization and fibrosis by inhibiting the activation of the NF-*κ*B/Snail signaling pathway, as summarized in Figure 8. This highlights the potential use of PFD in the treatment of AMD. However, further research is needed to explore how to maintain an appropriate balance between neovascularization and tissue fibrosis.

## Data Availability

The data that support the findings of this study are available from the corresponding author upon reasonable request.
